# *Helicobacter pylori* reinfection and its risk factors after initial eradication: A protocol for systematic review and meta-analysis

**DOI:** 10.1097/MD.0000000000025949

**Published:** 2021-05-14

**Authors:** Renliang Li, Ping Zhang, Ziyi Hu, Ying Yi, Lisha Chen, Hengyi Zhang

**Affiliations:** a Jiangxi University of Traditional Chinese Medicine; b The Affiliated Hospital of Jiangxi University of Traditional Chinese Medicine, Nanchang, Jiangxi Province, PR China.

**Keywords:** *Helicobacter pylori*, meta-analysis, protocol, reinfection, risk factors, systematic review

## Abstract

**Background:**

*Helicobacter pylori* (*H pylori*) infection is a common health problem, which is closely related to peptic ulcers, gastric cancer, and extragastric diseases. Drugs can successfully eradicate it. However, the recurrence of *H pylori* often occurs after initial eradication. To confirm the global incidence of *H pylori* reinfection and systematically evaluate its risk factors.

**Methods:**

We will search for the relevant literature through Chinese and English databases, with the retrieval deadline being November 2021. Databases include PubMed, Embase, Web of Science, the Cochrane Library, China National Knowledge Infrastructure, the Chongqing VIP Chinese Science and Technology Periodical Database, Wanfang Database, and China Biomedical Literature Database. Stata14.0 will be used to conduct this systematic review. The preferred reporting items for systematic reviews and meta-analysis protocols statements are followed in this protocol, and the PRISMA statement will be followed in the completed systematic review.

**Results:**

The results will be published in a peer-reviewed journal.

**Conclusions:**

This systematic review will provide evidence regarding the rate of *H pylori* reinfection and its risk factors after successful eradication. It can guide the management of patients with *H pylori* infection.

## 1 Introduction

Helicobacter pylori (*H pylori*), as we all know, is a notorious gastrointestinal microorganism, which can lead to chronic gastritis, peptic ulcer, and even evolve into gastric cancer.^[1]^ Besides, *H pylori* infection is also associated with extragastric diseases, especially cardiovascular, metabolic, and neurologic disorders.^[2,3]^ In the world, more than half of the human population is infected with *H pylori*.^[4]^ A meta-analysis^[5]^ found that a wide variation in the prevalence of *H pylori* for countries ranges from 18.9% (Switzerland) to 87.7% (Nigeria). Therefore, the eradication of *H pylori* has attracted much attention. *Kyoto Global Consensus Report*^[6]^ had stated that *H pylori* gastritis recommends eradication therapy for *H pylori*-infected individuals, except in the case of competing considerations. And it can effectively decrease the risk of gastric cancer by eradicating *H pylori*.^[7]^

The recurrence of *H pylori* infection can be classified into 2 distinct mechanisms: recrudescence and reinfection.^[8]^ The recrudescence is the reappearance of the original strain after initial eradication; it is considered to be a failure of eradication. The reinfection is infected with a new strain after initial eradication. The identification of *H pylori* strain requires the application of some molecular fingerprinting techniques. These techniques have very complicated procedures and require high personnel and facilities. So, at present, it cannot be widely used in clinical and scientific research. Some investigators believe that the recurrence of *H pylori* infection less than 1 year after eradication is classified as recrudescence, while reinfection if it was more than 1 year.^[9,10]^ Other researchers think that the change of *H pylori* from negative to positive at 1 year after initial eradication may be caused by recurrence or reinfection, and reinfection was the leading cause of recurrence at 3 years after successful eradication.^[11]^

Hu et al^[12]^ revealed that the global annual recurrence, reinfection, and recrudescence rate of *H pylori* were 4.3%, 3.1%, and 2.2%, respectively. In recent years, many studies have studied the mechanism and influencing factors of the recurrence of *H pylori*. There are significant differences in the rate of *H pylori* reinfection and recrudescence and risk factors in different countries and regions. Thus, this review intends to analyze the risk effects of *H pylori* reinfection by evidence-based medicine.

## 2 Methods

### 2.1 Protocol and registration

This protocol has been registered on the INPLASY website, and its registration number is INPLASY202140121, which could be accessed on https://inplasy.com/inplasy-2021-4-0121/.

### 2.2 Literature search

This systematic review and meta-analysis will be performed and reported according to the Preferred Reporting Items for Systematic Reviews and Meta-Analyses (PRISMA) guidelines.^[13]^ We will search the following databases from their inception to November 2021: PubMed, Embase, Web of Science, the Cochrane Library, China National Knowledge Infrastructure, the Chongqing VIP Chinese Science and Technology Periodical Database, Wanfang Database, and China Biomedical Literature Database. The following phrase: *Helicobacter pylori*, *Campylobacter pylori*, *H pylori*, *HP*, and recurrence, recurrences, recrudescence, recrudescences, relapse, relapses, recurrent, recurred, reinfection, re-infect∗, relapse∗ will be used for each electronic search. The search was restricted to human studies and literature written in English and Chinese. A strategy details for PubMed are shown in Table 1, and the Chinese databases will use these items translated by Chinese. At the same time, we will supplement and obtain the relevant literature, though searching manually the references included in the study. The electronic search will be conducted by 2 reviews (YY, LC) independently.

**Table 1:** PubMed search strategy.

Number	Search terms
#1	*Helicobacter pylori* [Title/Abstract] OR *Campylobacter pylori* [Title/Abstract] OR *H pylori* [Title/Abstract] OR *Hp* [Title/Abstract]
#2	recurrence [Title/Abstract] OR recurrences [Title/Abstract] OR recrudescence [Title/Abstract] OR recrudescences [Title/Abstract] OR relapse [Title/ Abstract] OR relapses [Title/Abstract] OR recurrent [Title/Abstract] OR recurred [Title/Abstract] OR reinfection [Title/Abstract] OR re-infect^∗^ [Title/Abstract] OR relapse^∗^ [Title/Abstract]
#3	#1 and #2

### 2.3 Study selection

#### 2.3.1 Definitions

- Successful eradication: Negative *H pylori* status in a previously *H pylori*-infected patient at least 4 weeks after initial eradication.
- *H pylori* recrudescence: A recrudescence, with the same strain occurred, during the 6 to 12 months period immediately after successful eradication.^[14]^
- *H pylori* reinfection: A recurrence with a new strain, which was negative for 12 months after successful eradication, becomes positive again at a later stage, based on both clinical and molecular evidence previously reported.^[15]^

#### 2.3.2 Inclusion criteria

The study is considered qualified when the following criteria are met.

1. Cohort study or cross-sectional study;
2. Patients with initial *H pylori* infection were successfully eradicated;
3. Age between 18 and 65 years old;

#### 2.3.3 Exclusion criteria

1. A follow-up time after successful eradication of fewer than 12 months;
2. Republished literature;
3. Research on insufficient data or lack of access to the full text;
4. Animal or cell experiments, reviews, meta-analyses, and conference presentations.

### 2.4 Data extraction and management

**Figure 1:**
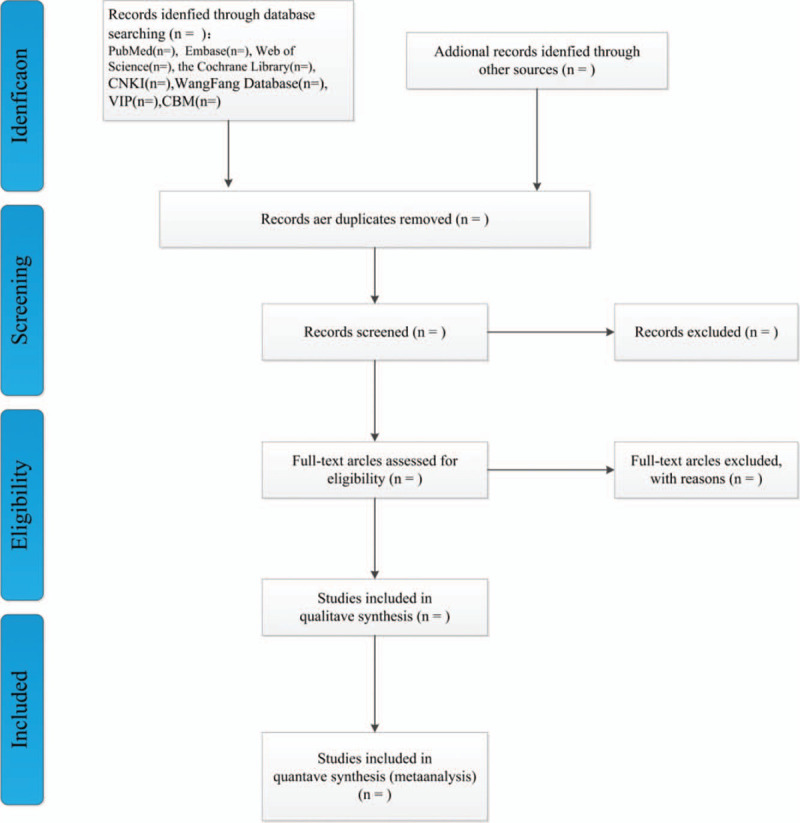
Flow diagram of literature retrieval.

Two authors (HZ and PZ) search and screen the literature independently. Provided that the 2 reviewers have different opinions, whether or not the literature should be included, it is advisable for them to resolve by discussion. We manage the search results through NoteExpress 3.3.0 software. The selection will be performed according to the PRISMA flow chart shown in Figure 1. The content of the data extraction: author's name, year of publication, title, country, The enforcement time of the study, study design, sample size, follow-up, eradication treatment, annual reinfection rate, multivariable adjusted hazard ratio or risk ratio, with their corresponding 95% confidence interval, and adjustment for confounding factors.

### 2.5 Risk of bias assessment

Newcastle-Ottawa Scale was applied to examine the methodological quality of the included studies.^[16]^ Newcastle-Ottawa Scale had: 4 items for study subjects (4 points), 1 item for intergroup comparability (2 points), and 3 items for result measurement (3 points), with a total score of 9 (http://www.ohri.ca/programs/clinical_epidemiology/oxford.asp).

### 2.6 Statistical analysis

This meta-analysis will be conducted using STATA 14.0. A random-effects model will be used to estimate the pooled reinfection rate and its risk factors with 95% confidence interval. Heterogeneity will be assessed using a chi-square test and *I*^*2*^ statistics (*P* value <.10 or *I*^*2*^ over 50% were defined as substantial heterogeneity). Publication bias will be estimated by the Begg test^[17]^ and Egger test,^[18]^ with *P* < .1 indicating statistically significant.

### 2.7 Sensitivity analysis

We will use the leave-one-out method for sensitivity analysis to judge the stability of outcome indicators.

### 2.8 Subgroup analysis

If the source of heterogeneity cannot be found after sensitivity analysis, we will do further subgroup analysis.

### 2.9 Ethics and dissemination

In this study, no individual data from participants will be involved, so ethics approval is not required.

## 3 Discussion

*H pylori* is a pathogen that can cause chronic and progressive gastric mucosal damage and is closely related to peptic ulcer, gastric cancer, gastric atrophy, gastric mucosa-associated tissue lymphoma, proliferative gastric polyp, and idiopathic thrombocytopenic purpura.^[6,19,20]^ It has been confirmed that *H pylori* eradication after treatment reduced the risk of gastric cancer.^[21]^ Recurrence either as recrudescence (inadequate treatment) and reinfection eliminates the benefits of *H pylori* eradication.

The recurrence of *H pylori* is related to lousy family personal living habits and re-exposure caused by exposure to *H pylori* infection. *H pylori* are transmitted through oral-oral, fecal-oral, and gastro-oral.^[8]^*H pylori* infection has familial aggregation. In daily life, those who were successful in *H pylori* eradication had close contact with family members infected with *H pylori*, shared meals, shared dental brushing tools, and came into contact with contaminated water and food. Any re-exposure of *H pylori* will cause recurrence. Zhou et al^[22]^ discovered that exposure to *H pylori* infectors, peptic ulcer, and hospitalization were its risk factors. In China, the annual *H pylori* reinfection rate was 1.5% per person-year and independently associated with the following risk factors: minority groups, the education at lower levels, a family history of gastric cancer, and the residence located in Western and Centra China.^[14]^

In developing countries, with a large population and high *H pylori* infection rates, it is essential to prevent *H pylori* infectious diseases, that is, to remain *H pylori*-negative for a long time. The purpose of this article is to analyze the risk factors of reinfection after *H pylori* successful eradication, to provide suggestions for clinical *H pylori* eradication treatment and management of recurrence after initial eradication.

## Author contributions

**Conceptualization:** Renliang Li and Ziyi Hu.

**Data curation:** Ying Yi, and Lisha Chen.

**Formal analysis:** Hengyi Zhang and Ping Zhang.

**Investigation:** Renliang Li and Ziyi Hu.

**Methodology:** Ping Zhang, and Ying Yi.

**Software:** Lisha Chen, and Ping Zhang.

**Supervision:** Ziyi Hu and Renliang Li.

**Writing – original draft:** Renliang Li, Ping Zhang, Ying Yi and Ziyi Hu.

**Writing – review & editing:** Renliang Li, Ping Zhang, and Lisha Chen.

## References

[1] Tao ZH Han JX Fang JY. Helicobacter pylori infection and eradication: exploring their impacts on the gastrointestinal microbiota. Helicobacter 2020;25:e12754. 32876377 10.1111/hel.12754

[2] Pellicano R Ianiro G Fagoonee S. Review: extragastric diseases and Helicobacter pylori. Helicobacter 2020;25: suppl 1: e12741. 32918343 10.1111/hel.12741

[3] Doheim MF Altaweel AA Elgendy MG. Association between Helicobacter pylori infection and stroke: a meta-analysis of 273,135 patients. J Neurol 2020;Online ahead of print. 10.1007/s00415-020-09933-x 32447554

[4] Ailloud F Didelot X Woltemate S. Within-host evolution of Helicobacter pylori shaped by niche-specific adaptation, intragastric migrations and selective sweeps. Nat Commun 2019;10:2273. 31118420 10.1038/s41467-019-10050-1 PMC6531487

[5] Hooi JKY Lai WY Ng WK. Global prevalence of *Helicobacter pylori* infection: systematic review and meta-analysis. Gastroenterology 2017;153:420–9. 28456631 10.1053/j.gastro.2017.04.022

[6] Sugano K Tack J Kuipers EJ. Kyoto global consensus report on Helicobacter pylori gastritis. Gut 2015;64:1353–67. 26187502 10.1136/gutjnl-2015-309252 PMC4552923

[7] Ford AC Yuan Y Forman D. Helicobacter pylori eradication for the prevention of gastric neoplasia. Cochrane Database Syst Rev 2020;7:CD005583. 32628791 10.1002/14651858.CD005583.pub3 PMC7389270

[8] Sjomina O Pavlova J Niv Y. Epidemiology of Helicobacter pylori infection. Helicobacter 2018;23: suppl 1: e12514. 30203587 10.1111/hel.12514

[9] Khor CJ Fock KM Ng TM. Recurrence of Helicobacter pylori infection and duodenal ulcer relapse, following successful eradication in an urban east Asian population. Singapore Med J 2000;41:382–6. 11256345

[10] Zhou G. Helicobacter pylori recurrence after eradication therapy in Jiangjin District, Chongqing. China Gastroenterol Res Pract 2020;2020:7510872. 32328098 10.1155/2020/7510872 PMC7165334

[11] Xue Y Zhou LY Lu HP. Recurrence of Helicobacter pylori infection: incidence and influential factors. Chin Med J (Engl) 2019;132:765–71. 30897591 10.1097/CM9.0000000000000146 PMC6595863

[12] Hu Y Wan JH Li XY. Systematic review with meta-analysis: the global recurrence rate of Helicobacter pylori. Aliment Pharmacol Ther 2017;46:773–9. 28892184 10.1111/apt.14319

[13] Liberati A Altman DG Tetzlaff J. The PRISMA statement for reporting systematic reviews and meta-analyses of studies that evaluate health care interventions: explanation and elaboration. PLoS Med 2009;6:e1000100. 19621070 10.1371/journal.pmed.1000100 PMC2707010

[14] Xie Y Song C Cheng H. Long-term follow-up of Helicobacter pylori reinfection and its risk factors after initial eradication: a large-scale multicentre, prospective open cohort, observational study. Emerg Microbes Infect 2020;9:548–57. 32160805 10.1080/22221751.2020.1737579 PMC7144303

[15] Zhang YY Xia HH Zhuang ZH. Review article: ‘true’ re-infection of Helicobacter pylori after successful eradication—worldwide annual rates, risk factors and clinical implications. Aliment Pharmacol Ther 2009;29:145–60. 18945250 10.1111/j.1365-2036.2008.03873.x

[16] Stang A. Critical evaluation of the Newcastle-Ottawa scale for the assessment of the quality of nonrandomized studies in meta-analyses. Eur J Epidemiol 2010;25:603–5. 20652370 10.1007/s10654-010-9491-z

[17] Begg CB Mazumdar M. Operating characteristics of a rank correlation test for publication bias. Biometrics 1994;50:1088–101. 7786990

[18] Egger M Davey Smith G Schneider M. Bias in meta-analysis detected by a simple, graphical test. BMJ 1997;315:629–34. 9310563 10.1136/bmj.315.7109.629 PMC2127453

[19] Sipponen P. Natural history of gastritis and its relationship to peptic ulcer disease. Digestion 1992;51: suppl 1: 70–5. 1397747 10.1159/000200919

[20] Graham DY. Helicobacter pylori update: gastric cancer, reliable therapy, and possible benefits. Gastroenterology 2015;148:719–31. 25655557 10.1053/j.gastro.2015.01.040 PMC4375058

[21] Kumar S Metz DC Ellenberg S. Risk factors and incidence of gastric cancer after detection of helicobacter pylori infection: a large cohort study gastroenterology. Gastroenterology 2020;158:527–36. 31654635 10.1053/j.gastro.2019.10.019 PMC7010558

[22] Zhou LY Song ZQ Xue Y. Recurrence of Helicobacter pylori infection and the affecting factors: a follow-up study. J Dig Dis 2017;18:47–55. 28026906 10.1111/1751-2980.12440

